# Tumor Segmentation in Colorectal Ultrasound Images Using an Ensemble Transfer Learning Model: Towards Intra-Operative Margin Assessment

**DOI:** 10.3390/diagnostics13233595

**Published:** 2023-12-04

**Authors:** Freija Geldof, Constantijn W. A. Pruijssers, Lynn-Jade S. Jong, Dinusha Veluponnar, Theo J. M. Ruers, Behdad Dashtbozorg

**Affiliations:** 1 Image-Guided Surgery, Department of Surgical Oncology, Netherlands Cancer Institute, Plesmanlaan 121, 1066 CX Amsterdam, The Netherlands; 2 Department of Nanobiophysics, University of Twente, Drienerlolaan 5, 7522 NB Enschede, The Netherlands

**Keywords:** colorectal cancer surgery, ultrasound, tumor segmentation, margin assessment, data scarcity, transfer learning, ensemble learning, image-guided surgery

## Abstract

Tumor boundary identification during colorectal cancer surgery can be challenging, and incomplete tumor removal occurs in approximately 10% of the patients operated for advanced rectal cancer. In this paper, a deep learning framework for automatic tumor segmentation in colorectal ultrasound images was developed, to provide real-time guidance on resection margins using intra-operative ultrasound. A colorectal ultrasound dataset was acquired consisting of 179 images from 74 patients, with ground truth tumor annotations based on histopathology results. To address data scarcity, transfer learning techniques were used to optimize models pre-trained on breast ultrasound data for colorectal ultrasound data. A new custom gradient-based loss function (GWDice) was developed, which emphasizes the clinically relevant top margin of the tumor while training the networks. Lastly, ensemble learning methods were applied to combine tumor segmentation predictions of multiple individual models and further improve the overall tumor segmentation performance. Transfer learning outperformed training from scratch, with an average Dice coefficient over all individual networks of 0.78 compared to 0.68. The new GWDice loss function clearly decreased the average tumor margin prediction error from 1.08 mm to 0.92 mm, without compromising the segmentation of the overall tumor contour. Ensemble learning further improved the Dice coefficient to 0.84 and the tumor margin prediction error to 0.67 mm. Using transfer and ensemble learning strategies, good tumor segmentation performance was achieved despite the relatively small dataset. The developed US segmentation model may contribute to more accurate colorectal tumor resections by providing real-time intra-operative feedback on tumor margins.

## 1. Introduction

Colorectal cancer is the third-most-commonly diagnosed cancer worldwide [1,2]. Treatment of colorectal cancer often involves surgery. Adequate surgical tumor resection, with a sufficient margin of healthy tissue both around and distally to the tumor, is crucial for patient prognosis. However, tumor boundary identification, especially during rectal cancer surgery, can be challenging without any intra-operative imaging guidance. The current golden standard for resection margin evaluation based on pathological examination takes place days after surgery. Unfortunately, a positive resection margin is still found in Dutch hospitals in 4.7% and 12.7% of T1-3 and T4 rectal tumors, respectively, worsening patient prognosis and warranting an improved method for intra-operative margin assessment [3].

Several methods have been developed for intra-operative colorectal tumor localization, such as barium enemas [4,5], frozen sections [6,7], pre-operative endoscopic tattooing or clip placement [8,9,10,11,12,13,14], intra-operative endoscopy [15], fluorescence guidance [16,17,18], and surgical navigation technology [19,20]. However, these techniques all have certain drawbacks, including radiation exposure, a long duration, non-optimal accuracy, complexity, and the need for special instruments or technical support. On the other hand, all the aforementioned methods are mainly focused on general tumor localization and are not capable of providing any information about the tumor boundaries. There is a lack of a technique that is precise, non-invasive, and simple and that provides real-time automatic analyses for intra-operative margin assessment. An intra-operative tumor margin assessment technique can provide real-time feedback to the surgeon, thereby potentially reducing the number of positive resection margins and improving patient outcomes.

Ultrasound (US) is a non-invasive and real-time imaging technique, which is widely used in health care for various purposes. The ability to visualize tissue structures could potentially aid in distinguishing healthy and tumor tissue during colorectal cancer surgery. Unfortunately, to the best of our knowledge, no studies have been performed on the use of intra-operative ultrasound (IOUS) for real-time resection margin assessment during colorectal cancer surgery. Mainly, endorectal ultrasound imaging is used prior to surgery to determine the tumor stage [21,22,23,24,25,26,27,28,29,30,31]. In most cases, the images are assessed by an expert radiologist. A recent study by Song et al. developed a deep neural network to distinguish endorectal ultrasound images from benign and malignant tumors [31]. Besides pre-operative staging, a couple of case and feasibility studies have used ultrasound imaging during colorectal cancer surgery to localize the tumor region, with or without the aid of pre-operative endoscopic clip placement [32,33,34,35,36,37,38,39]. However, this concerned rough localizations based on human interpretation of the US images, which is often challenging and requires training and experience.

Deep-learning-based approaches could facilitate the automatic analysis of colorectal ultrasound images during surgery. Convolutional neural networks (CNNs) are widely used in medical image analysis for image interpretation through automatic classification and segmentation. Many studies have already successfully developed automatic tumor segmentation networks for US imaging [40,41,42,43,44,45]. However, in the existing literature, these developments have primarily concentrated on specific oncological domains such as breast cancer and liver cancer. The application of deep learning techniques for automatic tumor segmentation in colorectal US images has thus far remained a largely unexplored area. In response, this study aimed to develop a network for automatic tumor segmentation in colorectal US imaging. In particular, we will focus on the application in open and laparoscopic colorectal cancer surgery, where the transducer makes direct contact with the resection plane on the outer surface of the colon, in contrast to prior studies on endorectal US imaging of colorectal cancer.

CNNs require a plethora of labeled data samples for adequate training, which is often scarce in the medical field, especially in the case of colorectal cancer, for which abdominal ultrasound is not routinely used in standard clinical care. Consequently, no public (labeled) datasets are available either. To overcome this issue, transfer learning techniques could be used. Pre-training models with comparable data can decrease the amount of data needed since the model does not need to be trained from scratch [46]. These pre-trained models can then be optimized using the dataset of the desired application. Therefore, this study will present a deep learning framework for tumor segmentation in colorectal US images, using pre-trained CNNs for tumor segmentation in breast US images. Although an individual neural network can show good performance, still an inherent bias may always be present in the model architecture [47]. Ensemble deep learning combats data scarcity by combining different model predictions into one final prediction. Aggregating multiple predictions can negate the errors of individual models, thus yielding a higher overall accuracy and improved generalization ability.

This study will present a deep learning framework for tumor segmentation in colorectal US images, using pre-trained CNNs for tumor segmentation in breast US images and ensemble learning techniques, to provide real-time intra-operative guidance on resection margins. The novel contributions of this paper can be summarized as follows:

- We acquired the first annotated extraluminal US dataset for colorectal cancer, with ground truth tumor annotation-based histopathology results.
- To combat data scarcity, we used transfer learning techniques to optimize models pre-trained on breast US data for colorectal US data.
- We applied ensemble learning methods to enhance overall tumor segmentation accuracy.
- We developed a new custom loss function (GWDice), focusing on the clinically relevant top margin of the tumor.
- We present the first study on automatic colorectal US segmentation for real-time intra-operative margin assessment.

The remainder of this paper is organized as follows: Section 2 starts with a description of the data acquisition, labeling, and pre-processing, after which the transfer and ensemble deep learning techniques that were used to develop a tumor segmentation network are discussed. The results are presented in Section 3, which is followed by the discussion and conclusion in Section 4 and Section 5.

## 2. Materials and Methods

In this section, we will first describe the workflow of colorectal ultrasound image collection and corresponding tumor annotations based on histopathology results. Then, the necessary image pre-processing steps will be performed, after which we will use transfer learning to optimize the models pre-trained on breast ultrasound data for our colorectal ultrasound dataset. The added value of using a custom gradient-based loss function and ensemble learning techniques will be examined. Finally, the tumor margin prediction performance will be assessed.

### 2.1. Data Acquisition

Freshly excised colorectal cancer specimens from 74 patients undergoing surgery at Antoni van Leeuwenhoek Hospital-Netherlands Cancer Institute (AvL-NKI) for colorectal cancer between April 2019 and April 2022 were included in this study, under the approval of the Hospital Ethics Review Board. Patients were included when diagnosed with a tumor of at least stage T2 in the colon, sigmoid, or rectum, based on pre-operative examinations. Both patients treated with and without neoadjuvant therapy were included in this study. No specific exclusion criteria were applied during the data-acquisition process, ensuring a good representation of the patient population and contributing to the generalizability of the study findings. All patients had given permission for the further use of their data and biological materials for scientific research.

**Figure 1:**
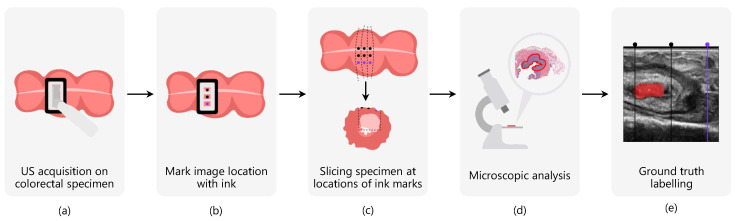
Data acquisition workflow: one to three US images are acquired on the freshly excised colorectal specimens, depending on the tumor size (**a**). Immediately after each acquisition, the measured imaging plane was marked with ink to allow correlation with histopathological results (**b**). During the histopathology process, the specimen is sliced at the locations of the ink marks (**c**), after which these slices are microscopically analyzed and the tumor is delineated by a pathologist (**d**). Based on these histopathological results and the location of the ink marks, the tumor was manually delineated in the acquired US image (**e**).

Ultrasound images were acquired using a Philips CX50 machine (Philips Research, Eindhoven, The Netherlands) and a Philips L15-7io high-frequency transducer (7–15 MHz), which was placed directly on the specimen’s surface. This transducer is well suited for high-resolution superficial measurements, with an imaging depth of only 3 cm. Per patient, 1 to 3 cross-sectional US images were acquired depending on the tumor size, which resulted in 179 US images in total (Figure 1a). The dataset was divided patient-wise into a training set of 121 images, a validation set of 28 images, and a test set of 30 images. The patient-wise division was implemented to enhance the generalizability of the network and prevent the potential introduction of bias related to similarities among images from the same patient, which could lead to overfitting to specific characteristics within patients. All images had a size of 430 × 344 px.

### 2.2. Data Labeling

Interpreting ultrasound images and annotating the tumor area can be challenging, even for an experienced radiologist. In order to obtain accurate labels, the measurement plane was marked with ink after the acquisition of each US image to correlate the data with the histopathology results (Figure 1b). To be able to retrieve the orientation during further processing, two black ink marks and one purple ink mark were placed.

After data acquisition, the specimens were brought to the pathology department for further processing according to standard protocols. Here, they were fixated in formalin for 48 h, after which the specimens were dissected into slices in such a way that each row of ink marks ended up in a separate slice. From these slices, the areas with ink marks on the surface were sampled in cassettes (Figure 1c). The final H&E-stained sections were digitally scanned for microscopic analysis (Figure 1d).

The tumor area was delineated by a pathologist in all digital slices. The black and purple ink marks, representing the exact US measurement locations, can be found back in the digital tissue slices. Similarly, the position of these locations in the US image is known, since the US probe was fixated using a mold during data acquisition and the same mold was used as a reference for marking the locations. Based on this correlation, the tumor was manually delineated in the acquired US image as the ground truth (Figure 1e).

### 2.3. Data Pre-Processing

Since this study was focused on resection margin assessment close to the specimen surface, the US images were cropped to the top half of the images and subsequently resized to 128 × 128 px. All images were normalized to a pixel intensity range of 0 to 1 before training the models.

During training, data augmentation was applied to generalize the models and reduce the risk of overfitting. The augmentation methods included vertical flipping, rotation, and gamma correction. Image rotation angles between −5 and 5 degrees were used. Gamma correction was applied according to the formula $P_{out}={\left(P_{in}\right)}^{\gamma }$, in which γ ranged from 0.8 to 1.2. These gamma values were based on the distribution of pixel intensities in the images.

### 2.4. Transfer Learning Using Pre-Trained Networks

Although deep learning models have proven very useful in medical image segmentation, they require a plethora of labeled data samples for adequate training, which is often scarce in the medical field. In order to combat data scarcity, a framework leveraging transfer learning is proposed.

Gomez-Flores et al. trained different convolutional neural network architectures for semantic segmentation of breast tumors in ultrasound images [40]. These publicly available models were trained on 3061 ultrasound images of different public datasets. We used five pre-trained models on breast US images as the foundation for a transfer learning approach with the colorectal ultrasound dataset acquired in this study; MobilenetV2 [48], Resnet18 [49], Resnet50 [49], U-net [50], and Xception [51].

The networks were initiated using the weights and biases that were acquired from pre-training on breast US data (no initialization). The transfer learning process involved further training these networks on batches of our colorectal US images. No layers were frozen during this process, allowing the entire network to undergo fine-tuning to our dataset. During the fine-tuning stage, learning rates were set a factor of 10 smaller than those of the original networks, ensuring the retention of previously learned features while adapting the weights to the new task. This approach effectively employed the pre-trained networks as a weight initialization scheme.

To optimize the performance, all network architectures were prototyped with hyperparameter tuning (learning rate, batch size, optimizers, dropout rates) through an exhaustive grid search. The best-performing network architecture was selected based on the highest Dice similarity coefficient. All training procedures were performed in MATLAB 2022a (MathWorks, Natick, MA, USA).

### 2.5. Loss Functions

#### 2.5.1. Generalized Dice Loss

Overlapping losses such as the Dice loss are commonly used to assess the context and shape of segmentation results. This loss is derived from the Dice similarity coefficient and divides the overlapping area of the ground truth mask and the predicted mask by the total area of both masks. To combat class imbalance between the foreground and background, a class-weighted variant of the Dice loss called the generalized Dice loss (GenDice) was used [52,53]. This adaptation of the Dice loss weighs the contribution of each class by the inverse of the area of the class in the ground truth mask, to counter the influence of larger regions on the Dice score; see Equation (1) and (2):

(1) $$GenDice\;loss=1-2\frac{\sum_{k=1}^{2}w_{k}\sum_{n}g_{kn}p_{kn}}{\sum_{k=1}^{2}w_{k}\sum_{n}g_{kn}+p_{kn}},$$

with *n* the number of pixels in the image, $g_{kn}$ the pixel values of the true tumor mask, $p_{kn}$ the pixel values of the predicted tumor mask, and $w_{k}$ the class weights for each class *k*:

(2) $$w_{k}=\frac{1}{{\left(\sum_{n=1}^{N}g_{kn}\right)}^{2}}.$$

#### 2.5.2. Gradient-Weighted Dice Loss

**Figure 2:**
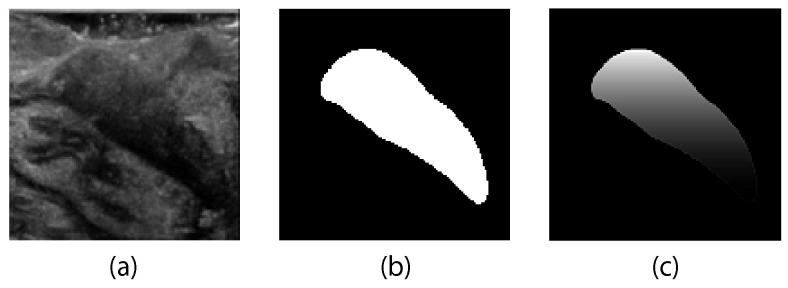
Gradient weighting of ground truth mask in the custom loss function. (**a**) Original US image, (**b**) ground truth mask as used in the GenDice loss function, and (**c**) gradient-weighted ground truth mask as used in the GWDice loss function.

Although resection margin assessment benefits from accurate tumor segmentation, the main clinical focus is the correct identification of the top margin of the tumor. Therefore, a custom loss function (GWDice) was developed in this study based on an expansion of the generalized Dice loss (GenDice). In order to emphasize the importance of the top border, weights were introduced that applied an exponential gradient in the vertical direction to the ground truth mask; see Figure 2. Weights were applied in a way that tumor weights at the bottom of the tumor stayed at the original weight assigned by the generalized Dice loss. Weights for the pixels at the top of the image were increased by a factor of two, which exponentially decreased to one over the height of the tumor. The method of calculating the gradient weighted ground truth mask is shown in Algorithm 1. The new GWDice loss function can be obtained based on this gradient weighted mask, as shown in Equation (3).

(3) $$GWDice\;loss=1-2\frac{\sum_{k=1}^{2}w_{k}\sum_{n}^{}g_{kn}t_{kn}p_{kn}}{\sum_{k=1}^{2}w_{k}\sum_{n}^{}t_{kn}+p_{kn}},$$

with *n* the number of pixels in the image, $g_{kn}$ the pixel values of the true tumor mask extracted from the image $I_{Gk}$, $t_{kn}$ the gradient-transformed pixel values of the true tumor mask extracted from the image $I_{Tk}$, $p_{kn}$ the pixel values of the predicted tumor mask, and $w_{k}$ the class weights for each class *k*.
**Algorithm 1** Gradient weighted ground truth mask.**Input:** Ground truth mask $I_{Gk}$, where k∈{1,2}     ▹Two-channel image: background (k=1) and foreground (k=2)**Output:** Gradient-weighted mask $I_{Tk}$1:**for all** (x,y) **do**                                                                                                                             ▹For all pixels in the image2:     $I_{T1}\left(x,y\right)=I_{G1}\left(x,y\right)$                                                                                                ▹Finding indexes of nonzero values3:     $M,N\leftarrow find(I_{G2}\left(x,y\right)==1)$4:     **if** $I_{G2}\left(x,y\right)$ is 1 **then**5:          $I_{T2}\left(x,y\right)\leftarrow {\left[\left(x-\max M\right)/\left(\max M-\min M\right)\right]}^{2}$                                          ▹Calculating the gradient weight6:     **else if** $I_{G2}\left(x,y\right)$ is 0 **then**7:        $I_{T2}\left(x,y\right)\leftarrow 0$8:     **end if**9:**end for**

### 2.6. Ensemble Learning

**Figure 3:**
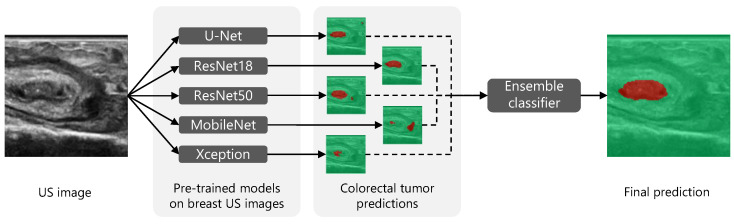
Visualization of the transfer and ensemble learning framework for tumor segmentation in colorectal US, using 5 models pre-trained on breast US data. Healthy tissue is highlighted in green; tumor tissue is highlighted in red.

Combining segmentation predictions from different models may reduce the outlying errors of single models. Hence, after retraining the five individual networks, their segmentation outputs were combined to further improve the final segmentation performance (see Figure 3). Multiple fusion strategies were examined: (1) unweighted averaging, in which the output probabilities of the five individual models were averaged; (2) weighted averaging, in which the output probabilities of the five individual models were averaged after applying a specific weight to each individual model; (3) voting, in which a tumor label was assigned when at least a certain number of individual models predicted tumor; and (4) an ensemble classifier, in which a pixel-based classification model was trained using the output probabilities of the five individual models as the input. For each fusion strategy, the weights and/or thresholds were optimized to achieve the best-possible combination of the Dice score and tumor margin error.

### 2.7. Post-Processing

Since tumors generally consist of one continuous area, without gaps, two post-processing steps were performed on the resulting tumor segmentation masks from all individual models and ensemble methods. First, the largest connected component was selected, to remove any separate small regions from the segmentation. Next, morphological closing was performed with a disk-shaped structuring element with a radius of 3 px to fill any remaining holes in the segmentation.

### 2.8. Performance Measures

**Figure 4:**
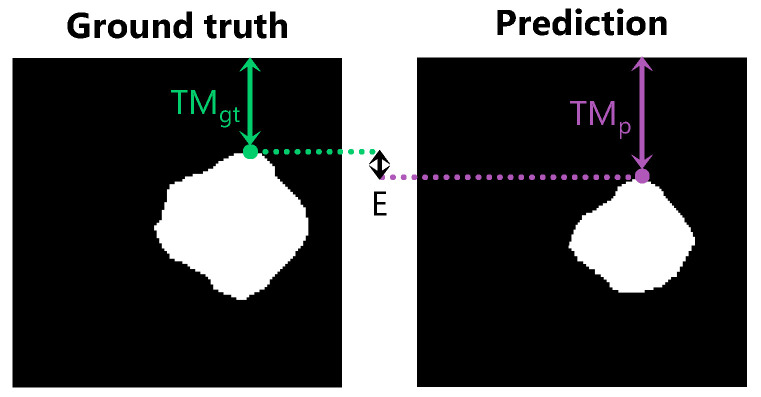
Visualization of the tumor margin error metric. TM${}_{\mathrm{gt}}$ shows the ground truth tumor margin; TM${}_{p}$ shows the predicted tumor margin. The tumor margin error, E, is the absolute difference between the ground truth and predicted tumor margin (in millimeters).

The performance of all network architectures was evaluated using the Dice similarity coefficient, the tumor margin error, and the area under the curve (AUC). The Dice similarity coefficient is a commonly used performance metric in medical image segmentation, measuring the amount of overlap between two segmentation masks (ranging from 0 to 1). A tumor margin error metric was devised to assess the accuracy of the top tumor margin prediction, which is calculated as the vertical distance between the top tumor pixel in the ground truth mask and the top tumor pixel in the predicted mask (in millimeters); see Figure 4. For comparing the performance, the average tumor margin error was calculated for all test images. The AUC represents the area under the receiver operating characteristic (ROC) curve, measuring the performance of the model irrespective of what classification threshold is chosen (ranging from 0 to 1). For the Dice and AUC, a larger value indicates better performance, while for the tumor margin error, a smaller value indicates better performance.

## 3. Results

In the coming subsection, first, the patient and tumor characteristics are summarized, after which the added value of using transfer learning, a custom-gradient-based loss function, and ensemble learning for tumor segmentation in colorectal US images will be examined successively. Finally, the resection margin prediction performance will be assessed.

### 3.1. Patient and Tumor Characteristics

Ultrasound images were obtained from freshly excised specimens of 74 colorectal cancer patients, of whom 41% received neoadjuvant therapy. Tumors with varying locations and T-stages were included, with the majority situated in the rectum and classified as stage pT3. The mean tumor diameter measured 4.1 ± 1.9 cm, with an average margin to the tumor of 6.4 ± 3.7 mm. A detailed breakdown of the patient and tumor characteristics is presented in Table 1.

### 3.2. Comparison between Scratch Training, Pre-Trained Models, and Transfer Learning

**Figure 5:**
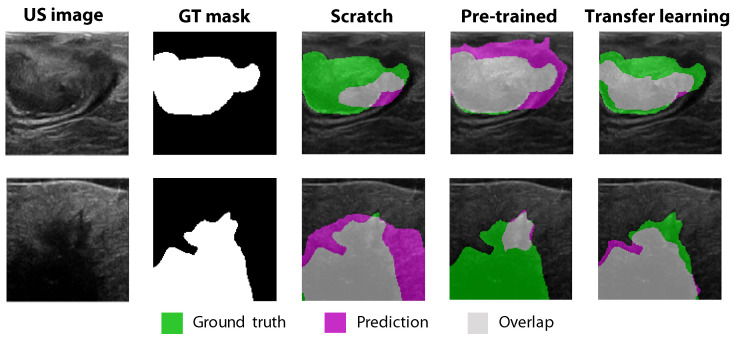
Visualization of tumor segmentations when training the individual networks from scratch, when using the networks that were pre-trained on breast US data, and after transfer learning of the pre-trained models with our colorectal US dataset. All results were obtained for the individual Resnet50 network, using the standard GenDice loss function.

To examine the impact of transfer learning on the colorectal tumor segmentation performance, the Dice similarity coefficient was initially calculated under two conditions: by training individual networks from scratch and by using the networks that were pre-trained on breast US data. These results were compared to the performance after transfer learning the pre-trained models with our colorectal data, which is summarized in Table 2. A clear increase in segmentation performance can be seen when the pre-trained networks were re-trained with our colorectal dataset. Using transfer learning, an average Dice coefficient of 0.78 was reached over all networks, while with training from scratch and the pre-trained breast US models, mean Dice coefficients of 0.68 and 0.63 were reached, respectively. Examples of the segmentation differences between the three methods are visualized in Figure 5. The figure shows that training from scratch using the pre-trained breast US models resulted in much over- and under-segmentation in our colorectal US dataset, depending on the situation. After retraining the pre-trained models with our colorectal US dataset (transfer learning), the tumor was segmented more consistently and accurately.

### 3.3. Comparison between GenDice and GWDice Loss Functions

**Figure 6:**
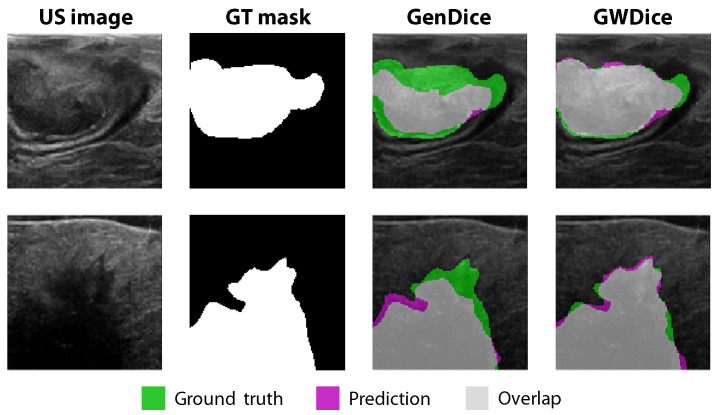
Visualization of tumor segmentations using the standard GenDice loss function and the custom GWDice loss function, for the individual Resnet50 network.

The performance of the custom GWDice loss function (introduced in Section 2.5.2) after transfer learning was compared to the commonly used GenDice loss function for all five individual models; see Table 3. The GWDice loss function slightly increased the mean Dice score from 0.78 to 0.80, while the mean tumor margin prediction error clearly decreased from 1.08 mm to 0.92 mm. This showed that the custom loss function was able to improve the tumor margin prediction by emphasizing the top margin of the tumor, without compromising the segmentation of the overall tumor contour. Examples of the segmentation differences between both loss functions are visualized in Figure 6. The biggest differences between the two loss functions can be seen at the top border of the tumor mask.

### 3.4. Comparison between Individual Models and Ensemble Learning

**Figure 7:**
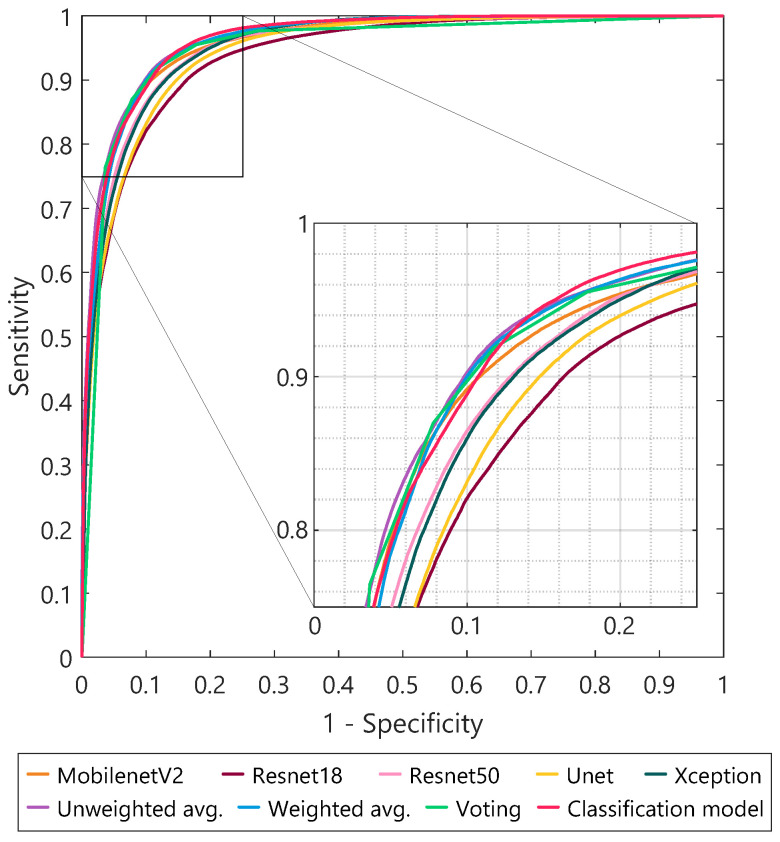
Receiver operating characteristic (ROC) curves of tumor segmentation for both the individual models and the different ensemble methods.

**Figure 8:**
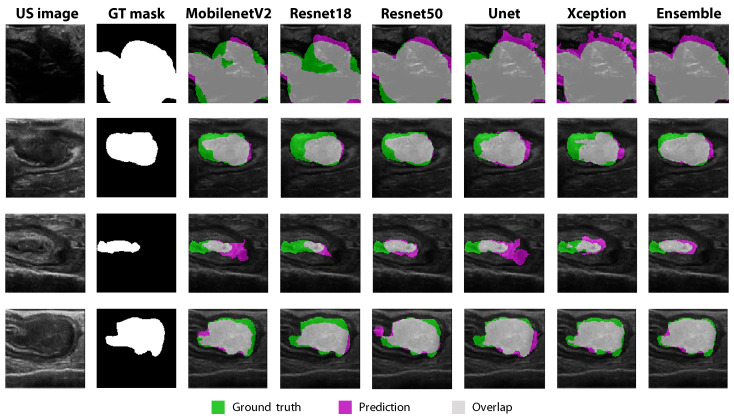
Visualization of tumor segmentations for four different tumor samples (rows 1–4), using the five individual models (column 3–7), and the classification ensemble method (column 8). For all results, transfer learning with the GWDice loss function was used.

The tumor segmentation performances of the different ensemble strategies are shown in Table 4, together with the average performance of the individual models. For all results, transfer learning with the GWDice loss function was used. Combining the tumor segmentation predictions from individual models into one ensemble prediction further improved the Dice coefficient from a mean of 0.80 to 0.84, and the tumor margin prediction error decreased from a mean of 0.92 mm to 0.67 mm. Between the different ensemble strategies, no major differences can be seen. The corresponding ROC curves are shown in Figure 7, for all individual models and ensemble methods. The highest AUC values were reached using the unweighted averaging (0.965) and classification ensemble methods (0.964), compared to an average AUC value of 0.951 for the individual segmentation models. The curves show that, by choosing an appropriate threshold value, a tumor segmentation sensitivity of 0.95 and specificity of 0.85 can be reached. Examples of the resulting tumor segmentations are visualized in Figure 8. Each individual segmentation model produced a slightly different tumor segmentation, with, at certain locations, over- or under-segmentation. Using the ensemble approach, the best of all these variations was combined into one final prediction, which showed the highest overlap with the ground truth tumor contour (column 8 in Figure 8).

As a reference, the tumor was annotated by two independent observers in ten randomly selected images to determine the human observers’ agreement on both the Dice coefficient and the tumor margin. This showed an inter-observer Dice score of 0.84 and tumor margin variation of 0.59 mm.

### 3.5. Resection Margin Prediction

**Figure 9:**
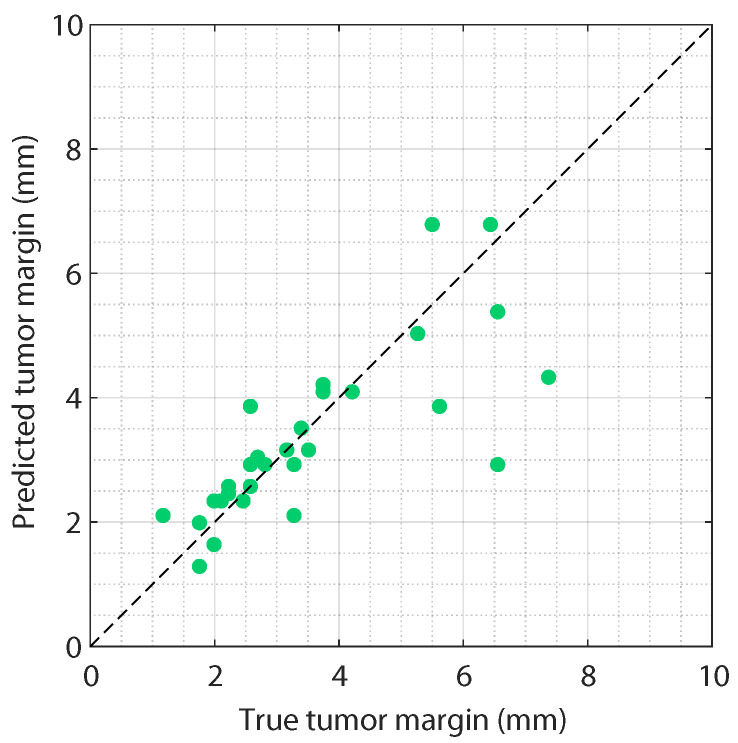
Predicted tumor margins versus the true tumor margins for all images in the test set, using the classification ensemble method.

The agreement between the predicted tumor margins using deep ensemble learning and the true tumor margins based on manual annotations is demonstrated in Figure 9, for all US images in the test set. This figure shows that small margins can be predicted with high accuracy. For tumor margins larger than 5 mm, some larger errors can be seen. A correlation coefficient of 0.77 was found, indicating a strong positive correlation between the true and predicted tumor margins. In addition, the tumor margin prediction results using our ensemble method did not show a consistent over- or under-estimation of the tumor margin.

### 3.6. Optimization

**Figure 10:**
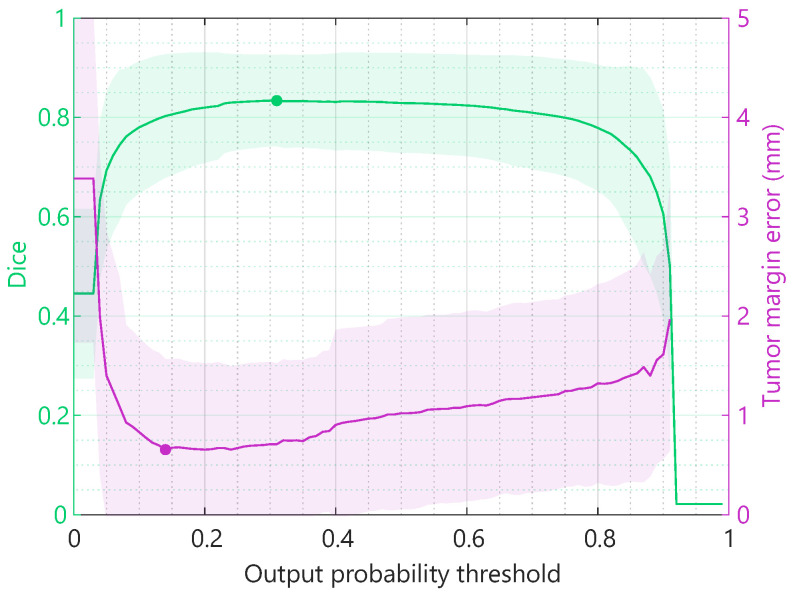
Dice and tumor margin error for every possible output probability threshold between 0 and 1, using the classification ensemble method. The solid line and shaded area represent the average and standard deviation of all images in the test set, respectively.

Choosing the optimal output probability threshold for the networks involves a trade-off between the best Dice coefficient and tumor margin error. The results presented in the previous section were based on a threshold for an optimal balance between the Dice and tumor margin error. However, when for the final application, one of them is most important, the thresholds can be further optimized for the Dice or tumor margin specifically. Figure 10 shows the resulting Dice and tumor margin error for the classification ensemble method, using every possible output probability threshold between 0 and 1. When optimizing purely the Dice coefficient, a maximum Dice of 0.84 can be achieved compared to the 0.83 previously reported using ensemble learning. When optimizing purely the tumor margin prediction error, a minimum tumor margin error of 0.64 mm can be achieved compared to the 0.67 mm previously reported using ensemble learning.

## 4. Discussion

In this paper, a model for automatic tumor segmentation in colorectal US images was developed, to provide real-time guidance on resection margins using intra-operative US. First, the added value of using transfer learning, a new custom gradient-based loss function, and ensemble learning was assessed, successively. Finally, the resection margin prediction accuracy based on the segmentation results was evaluated.

Due to data scarcity in the field of intra-abdominal colorectal ultrasound images, CNN models pre-trained for tumor segmentation in breast US were used as a starting point. After re-training these models with our colorectal US dataset, the segmentation performance (mean Dice of 0.78) outperformed the results that were achieved using only the pre-trained CNNs (mean Dice of 0.63) or training the CNN models from scratch (mean Dice of 0.68); see Table 2 and Figure 5. Although the pre-trained networks were trained on a large dataset of more than 3000 breast US images, it does concern a different application (breast tissue), which may explain why this method achieved the lowest Dice coefficient. On the other hand, training the CNNs from scratch was performed using our colorectal US dataset, which resulted in a slightly higher Dice coefficient. However, the fact that this dataset was relatively small may be a reason why optimal results were not achieved. Using the transfer learning method, the networks were pre-trained on a large comparable dataset, after which they were optimized on our current colorectal dataset. This strategy clearly increased the tumor segmentation performance. The available pre-trained models for tumor segmentation in breast US concern long-established neural network architectures. In follow-up research, more recent network architectures (i.e., EfficientNetV2 or ConvNeXt) could be investigated, where transfer learning techniques with a large comparable dataset may be used as well to combat data scarcity when training these networks from scratch.

A new custom GWDice loss function was introduced to put more emphasis on the top tumor border, which is clinically most important for intra-operative resection margin assessment. The new loss function achieved the desired effect: improving the top tumor margin prediction from 1.08 mm to 0.92 mm, while preserving a good general overlapping score (the mean Dice score increased from 0.78 to 0.80); see Table 3 and Figure 6. Currently, a vertical exponential gradient was used to adjust the weights in the ground truth mask. In future research, exploring other intensity profiles where the weights of the ground truth mask decrease less or more rigorously in the vertical direction could be interesting.

As a next step, multiple ensemble learning techniques were examined. The results showed that combining predictions from the five individual models further increased the Dice score from 0.80 to 0.84 and decreased the tumor margin error from 0.92 to 0.67 mm (Table 4 and Figure 8), compared to using one separate model. All four ensemble methods that were evaluated in this study resulted in comparable tumor segmentation performance metrics. As can be seen in Figure 8, the ensemble method canceled out some of the areas with over- and under-estimation that the different individual models showed. In this study, some commonly used ensemble strategies were used to examine whether ensemble learning could positively influence the segmentation performance. In the future, more sophisticated ensemble strategies could be explored as well.

To provide intra-operative guidance on resection margins, accurate tumor margin prediction is crucial. In this study, an average tumor margin prediction accuracy of 0.67 mm was achieved. This value is in the same order of magnitude as the US resolution (≈0.5 mm). The use of high-frequency ultrasound, with a higher spatial resolution and lower penetration depth, may achieve a slightly higher tumor margin prediction accuracy. In addition, the accuracy of 0.67 mm fell within the resection margin of 1 mm, which is generally used in colorectal cancer surgery. Figure 9 showed larger errors for deeper tumor margins, which could be due to a decrease in the US signal deeper in the tissue. However, given the fact that tumor detection close to the resection surface (up to 5 mm in depth) is clinically most relevant, this is not a major concern.

Although a correlation with histopathology was performed to obtain accurate tumor annotations, small errors might have been made during the manual annotations. The Dice coefficient of 0.84 achieved with our automatic segmentation model was equal to the human observers’ agreement of 0.84. In the case of the resection margin prediction, an accuracy of 0.67 mm was achieved with our automatic segmentation model, compared to an inter-observer variability of 0.59 mm. This shows that the automatic segmentation model achieved comparable results to the human observers, indicating that intra-operative US in combination with an automatic tumor segmentation model seems promising and may contribute to more accurate colorectal tumor resections.

Figure 10 shows that the optimal output probability threshold depends on the metric used to evaluate the performance of the segmentation model. The thresholds can be optimized specifically for the final application and user wishes, e.g., fully focused on achieving the best tumor margin, the entire tumor contour, or a combination. A similar trade-off can be made between sensitivity and specificity.

While our developed network demonstrated accurate tumor segmentations and tumor margin predictions for our colorectal US dataset, it is important to acknowledge certain limitations of our study. The use of a single US device and transducer introduces a potential challenge in generalizing our findings to other US devices and datasets, as hardware variations may impact tumor appearance and image quality. Future research should explore the generalizability of our model, including the custom loss function (GWDice), to diverse US devices and datasets (possibly even extending to other oncological fields). Potential hardware differences may be addressed by applying pre-processing steps, such as equalizing or enhancing the resolution and contrast. Additionally, the relatively small dataset size in our study may affect the model’s generalizability as well. Addressing this limitation by expanding the dataset in future research will contribute to a more-comprehensive evaluation of the proposed methods and their applicability across a broad patient population.

Currently, the US images were acquired on specimens from patients undergoing surgery for colorectal cancer, directly after surgical resection. The next step towards the clinical implementation of intra-operative US with automatic tumor segmentation would be to evaluate the tumor segmentation performance in vivo during surgery on intra-operatively acquired US images. Specifically, this in vivo validation should involve a comparison of the model’s predicted tumor margins during surgery with the definitive histopathological results obtained post-operatively. Subsequently, a randomized study could be considered to prove the clinical value of intra-operative US-based tumor segmentation in terms of positive margin incidence, patient outcomes, or surgery duration. The developed US segmentation model could contribute to more accurate colorectal tumor resections by providing real-time intra-operative feedback to surgeons on tumor margins. This may ultimately reduce the positive margin rate and enhance patient outcomes.

## 5. Conclusions

This study presented a deep learning framework for tumor segmentation in colorectal US images. A colorectal US dataset was acquired, with ground truth tumor annotations based on histopathology results. Using transfer learning to optimize the CNNs pre-trained on breast ultrasound data for the current colorectal ultrasound dataset, good tumor segmentation performance could be achieved despite the data scarcity. A new custom gradient-based loss function (GWDice) was developed, which emphasized the top margin of the tumor while training the networks. Ensemble learning methods were applied to combine the tumor segmentation predictions of multiple individual models and further improve the overall tumor segmentation performance, resulting in a final Dice coefficient of 0.84 and a tumor margin prediction accuracy of 0.67 mm. To the best of our knowledge, this is the first study on tumor segmentation for intra-operative colorectal US in the literature. Automatic tumor segmentation in colorectal US enables real-time intra-operative guidance and may contribute to more accurate colorectal tumor resections.

## Figures and Tables

**Table 1:** Patient and tumor characteristics.

Method	Number of Patients (%)
Gender	
Female	39 (53%)
Male	35 (47%)
Tumor location	
Colon	26 (35%)
Sigmoid	18 (24%)
Rectum	30 (41%)
Neoadjuvant therapy	
Yes	30 (41%)
No	44 (59%)
T-stage *	
pT1	2 (3%)
pT2	11 (15%)
pT3	44 (59%)
pT4	17 (23%)
Tumor diameter	4.1 ± 1.9 cm
Tumor margin	6.4 ± 3.7 mm

* T-stage based on the final histopathological results obtained after surgery.

**Table 2:** Tumor segmentation performance on the test set when training the individual networks from scratch, when using the networks that were pre-trained on breast US data, and after transfer learning of the pre-trained models with our colorectal US dataset. All networks were trained with the standard GenDice loss function.

Method	Dice		
	Scratch	Pre-Trained on Breast US	After Transfer Learning
MobilenetV2	0.70	0.65	0.76
Resnet18	0.72	0.65	0.77
Resnet50	0.70	0.63	0.78
U-net	0.59	0.55	0.79
Xception	0.69	0.67	0.80
Mean	0.68	0.63	0.78

**Table 3:** Tumor segmentation performance on the test set of the standard GenDice loss function compared to the custom GWDice loss function.

Method	Dice		Tumor Margin	
	GenDice	GWDice	GenDice	GWDice
MobilenetV2	0.76	0.80	1.03 mm	0.96 mm
Resnet18	0.77	0.79	1.25 mm	0.99 mm
Resnet50	0.78	0.81	1.26 mm	0.96 mm
U-net	0.79	0.79	0.83 mm	0.88 mm
Xception	0.80	0.81	1.02 mm	0.83 mm
Mean	0.78	0.80	1.08 mm	0.92 mm

**Table 4:** Tumor segmentation performance on the test set of individual models compared to multiple ensemble methods, using transfer learning with the GWDice loss function.

Method	Dice	Tumor Margin	AUC
Individual models (mean)	0.80	0.92 mm	0.95
Unweighted averaging	0.84	0.68 mm	0.97
Weighted averaging	0.84	0.68 mm	0.96
Voting	0.84	0.67 mm	0.95
Classification model	0.83	0.67 mm	0.97

## Data Availability

The data underlying the results presented in this paper are not publicly available, but may be obtained from the authors upon reasonable request.
